# The potential of FBC fly ashes to reduce CO_2_ emissions

**DOI:** 10.1038/s41598-020-66297-y

**Published:** 2020-06-11

**Authors:** Alicja Uliasz-Bocheńczyk, Eugeniusz Mokrzycki

**Affiliations:** 1 AGH University of Science and Technology, Faculty of Mining and Geoengineering, Mickiewicza 30, 30-059 Krakow, Poland; 2 Mineral and Energy Economy Research Institute of the Polish Academy of Sciences, Wybickiego 7A, 31-261 Krakow, Poland

**Keywords:** Climate sciences, Environmental sciences

## Abstract

The production of electricity and heat in Poland is the reason why the commercial power industry is the largest emitter of CO_2_. At the same time, significant amounts of solid by-products of combustion, which can be used to bind CO_2_ by mineral carbonation, are generated during the production processes. The article presents the results of research on mineral sequestration of CO_2_ (suspension-CO_2_) using fluidized bed combustion (FBC) fly ashes from hard coal combustion. The analyzed fluidized bed combustion (FBC) fly ashes were characterized by a significant free CaO content (1.7–6.8%) and a high CO_2_ binding potential ranging from 9.7 to 15.7%. In the case of fluidized bed combustion (FBC) fly ashes suspensions, the basic product of the carbonation process is calcium carbonate, which is clearly indicated by the results of the phase composition determination of solidified suspensions of fluidized bed combustion (FBC) fly ashes. The degree of carbonation, i.e. the degree of CO_2_ binding, calculated on the basis of the calcium carbonate content, in the analyzed suspensions was up to 1.1%. Mineral carbonation also reduces the leachability of pollutants such as: Zn, Cu, Pb, Ni, As, Hg, Cd, Cr, Cl, and SO_4_^2-^. The pH is also reduced from about 12 to about 9. Aqueous suspensions of fluidized bed combustion (FBC) fly ashes with introduced CO_2_ can potentially be used in underground mining. These activities are in line with the concepts of Carbon Capture and Utilization and the idea of circular economy.

## Introduction

The recent anthropogenic greenhouse gas emissions are the highest in history and are probably the main cause of the climate change observed since the mid-20th century - the so-called global warming. A particular role is played by CO_2_ emissions from fossil fuel combustion and industrial processes, which in the period from 1970 to 2010 accounted for approximately 78% of the total increase in greenhouse gas emissions^1^.

The commercial power industry is the largest emitter of carbon dioxide in Poland (Table 1). This is due to the fact that coal is the primary fuel used in the power industry, which is conditioned by the abundant resources of this energy source in Poland. Due to these conditions, it will be difficult to reduce CO_2_ emissions from energy production. One of the possibilities to reduce CO_2_ emissions is mineral sequestration using waste generated in the same energy production process.

**Table 1:** CO_2_ emission from coal combustion in professional power industry, Gg^3^.

Year	Total emission	Emission from hard coal combustion
2008	144,195	84,228
2009	144,227	87,301
2010	148,573	92,949
2011	150,188	91,019
2012	147,338	85,161
2013	146,947	60,513
2014	138,061	77,573
2015	142,120	81,843
2016	140,183	82,724

The mineral sequestration of CO_2_ is a method that, due to the stable binding of CO_2_, is an alternative to geological storage.

Mineral sequestration is a method of reducing CO_2_ emissions, in which fly ashes from coal combustion play a special role. They are produced in the same energy production process; mineral sequestration may allow their economic use.

The waste products which should primarily be taken into account when it comes to CO_2_ binding are the fly ashes with a high content of free CaO^2^.

Studies on the suitability of fly ashes from coal combustion for reducing CO_2_ emissions by mineral carbonation are carried out worldwide by different authors using different process conditions (Table 2).

**Table 2:** CO_2_ utilization using hard coal fly ashes.

Type of carbonation	References	Result of process
Suspension – CO_2_	^23^	carbonation efficiency of 83.5% ‒ final CO_2_ 3.2%, i.e. 32 g CO_2_/kg fly ashes
Suspension – CO_2_	^24^	79% carbonation efficiency
Aqueous carbon sequestration process	^25^	amorphous calcium carbonate
Aqueous carbonation	^26^	capacity to sequester CO_2_: 26 kg/CO_2_/Mg fly ashes
Accelerated mineral carbonation	^27^	3.86 ± 1.28 CaCO_3_
Solid – CO_2_	^28^	0.29−4.29 mmol CO_2_ capture/g fly ashes
Solid – CO_2_	^29^	CO_2_ uptake: 18.2 wt. %
Suspension – CO_2_	^30^	10.71−27.05 kg of CO_2_ per ton of fly ashes
Suspension – CO_2_	^31^	CaCO_3_ content – 2.27% CO_2_ absorption: 0.42–1.31 g CO_2_/100 g
Suspension – CO_2_	^32^	0.43–12.82% of CO_2_ binding CO_2_ absorption: 2.15–9.54 g CO_2_/100 g
Suspension – CO_2_	^5^	CO_2_ absorption: 1.4–8.8 g CO_2_/100 g
CO_2_ ‒ FA/brine slurry	^33^	CO_2_ sequestration potential: 36.47 and 71.84 kg of CO_2_/Mg fly ashes
Fly ashes brine dispersion – CO_2_	^34^	CO_2_ content – 2.75–6.5% wt.
Two-step indirect aqueous carbonation	^35^	CO_2_ sequestration: 0.008 kg of CO_2_/kg of fly ashes
Indirect mineral carbonation	^36^	CO_2_ storage capacity: 31.1 mg CO_2_/g FA

Unfortunately, the existing literature lacks information on the type of boilers, which directly affects the properties of by-products of coal combustion.

The analyses are carried out using fly ashes and water suspensions and dry fly ashes in direct and indirect carbonation.

FBC fly ashes are waste materials that, due to the high content of free CaO, can be used to reduce CO_2_ emissions by mineral carbonation. The high CaO content in fluidized ashes is due to the fact that the coal combustion process is integrated with the desulfurization process. Fluidized ashes contain, in addition to components derived from coal combustion, desulfurization products and sorbent residues.

Mineral carbonation studies carried out using fly ashes from combustion in fluidized bed boilers indicate the high potential of this waste (Table 3). However, there has been relatively little research focused on this topic. The work presented in this paper contributes to the research in this area by analyzing fly ashes from coal combustion.

**Table 3:** CO_2_ utilization using fluidized bed combustion (FBC) fly ashes.

Type of carbonation	References	Result of process
Solid – CO_2_	^37^	the maximum CO_2_ sequestration capacity: 60 g CO_2_/kg fly ashes max. sequestration efficiency: 28.74%
Suspension – CO_2_	^38^	CO_2_ sequestration: 1.27, 2.50% by mass
Sonochemical-enhanced carbonation	^39^	max. conversion to carbonate: 50.5%

The article presents the results of studies on the use of mineral sequestration using aqueous suspensions of fluidized bed combustion (FBC) fly ashes from hard coal combustion in order to reduce CO_2_ emissions.

The research results presented in the article are a new approach to the analysis of CO_2_ binding by aqueous fly ashes suspensions as a method of reducing the emission of this harmful greenhouse gas as well as allow the recovery of fly ashes from coal combustion in fluidized bed boilers. As shown in Table 3, little research focused on the use of fly ashes from fluidized bed boilers.

In an attempt to reduce pollution to the atmosphere, while retaining efficient fuel combustion, more and more power plants and heat and combined heat and power plants install fluidized bed boilers.

In recent years, the number of boilers in power plants and combined heat and power plants using fluidized bed combustion has increased. In 1998 in Poland, there were only two fluidized-bed boilers used in the commercial power industry, while there were already 11 in 2003 and 27 in 2016^3^.

In Poland, FBC fly ashes are now used mainly in underground mining, in the form of aqueous suspensions, and in road construction^4^.

Fly ashes from coal combustion in the form of water suspension have been used in underground mining since the 1980s e.g. for sealing underground voids^6^.

The use of FBC fly ashes for CO_2_ binding by mineral carbonation and depositing them in underground mines^5–7^ is in line with the idea of closed-loop recycling.

**Figure 1:**
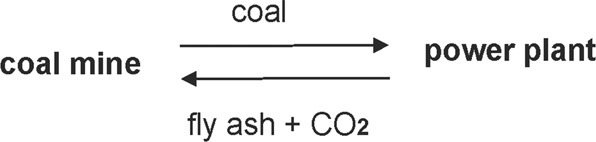
CO_2_ utilization in fly ashes suspension in coal mine.

As a result of the energy production process from coal extracted in mines are generated - fly ashes and CO_2_, which can potentially be used in suspension technology and deposited in mines^5–7^, thus closing the waste and CO_2_ circulation (Fig. 1).

It is the implementation of cleaner production technologies.

The mineral sequestration in fly ashes-water suspensions is a particularly interesting method because the basic product formed in the process - CaCO_3_ is a component of the hardened suspension. Therefore, fly ashes from the energy sector can be recovered, while CO_2_ can be disposed of, and thus the problem of using by-products of the combustion process – fly ashes is solved^5^.

However, the use of suspensions subjected to the mineral sequestration, which could potentially be used in mining, requires knowledge of the degree of binding of CO_2_ based on determining the increase in the amount of CaCO_3_ as the basic product of the carbonation process. Determining the amount of CaCO_3_ produced in the carbonation process allows estimating how much CO_2_ can be permanently bound in hardened suspensions and successfully used in underground mining technologies.

The problem of reducing CO_2_ emissions is also very important due to the increasing EUA prices, which in February 2017 stood at EUR 4.90 and in September 2019 at EUR 27.03^8^.

## Experimental

### Research methodology

FBC fly ashes used in the study were obtained from the combustion of aqueous suspensions with the following fly ashes to water weight ratios: PF 1−0.9; PF 2−0.6; PF 3–0.8; PF 4–0.7.

When determining the degree of mineral sequestration of CO_2_, the phase composition was tested using differential thermal analysis and the calcite content in the suspension was determined by the thermogravimetric analysis.

A complementary study on the leachability of pollutants from fly ashes-water suspensions was carried out in order to determine effects of their exposure to CO_2_. The suspensions were exposed to CO_2_ in installation consisting of research chambers, recording equipment, CO_2_ cylinders, and a regulator^5,7^.

The analysis of phase composition (DTA) and the analysis of calcium carbonate content (TG) were carried out in the atmosphere with the heating rate of 10 °C·min^−1^.

The microstructural analysis of fly ashes-water suspensions was carried out using a JEOL scanning electron microscope equipped with an Oxford Instruments EDS 540 system.

In determining the impact of CO_2_ on the properties of waste-aqueous suspensions, a series of tests was performed to determine the leachability of: chlorides (Cl^−^), sulfates (SO_4_^2−^), arsenic (As), chromium (Cr) (total), cadmium (Cd), copper (Cu), lead (Pb), nickel (Ni), zinc (Zn) and mercury (Hg), chemical oxygen demand (COD), and pH.

The content of arsenic, chromium, cadmium, copper, lead, nickel, zinc, arsenic, and mercury in aqueous solutions was analyzed using two methods: ICP AES and plasma mass spectrometry. The chemical oxygen demand (COD) was tested in accordance with the PN-74 C-04578/03 standard. Chloride content was determined using Volhard method and sulfate content using atomic emission spectrometry with inductively coupled plasma. The obtained results were compared with values defined in the PN-G-11011 standard: Materials for Backfilling and Caulking of Cavings – Requirements and Tests (due to the fact that fly ashes from fluidized bed boilers are primarily used in the mining industry).

### Materials used in the study

FBC fly ashes from coal combustion characterized by a high content of CaO and free CaO (Table 4) were selected for the analysis.

**Table 4:** The content of CaO and free CaO and maximum binding capacity of CO_2_,%.

Type of fly ashes	Content^7^		Maximum binding capacity of CO_2_
	CaO_total_	CaO_free_	
PF 1	15.5	4.7	15.7
PF 2	11.8	1.7	9.7
PF 3	19.5	6.8	12.2

The study used fly ashes (FBC fly ashes) from the following fluidized bed boilers:

- CFB boiler, hard coal-fired boiler, fluid circulation boiler, atmospheric furnace boiler, natural circulation boiler, and single drum boiler − PF 1,
- OF type, hard coal-fired, two-pass boiler, atmospheric furnace with circulating fluidized bed − PF 2,
- OF type, circulating fluidized bed − PF 3.

The analyzed fly ashes were characterized by high content of CaO and free CaO (Table 3).

For the analyzed fly ashes, the maximum capacity of binding was calculated using Steinour formula^9^:

1 $$C{O}_{2}({\rm{ \% }})=0.785({\rm{C}}{\rm{a}}{\rm{O}}-0.7{\rm{S}}{{\rm{O}}}_{3})+1.09{\rm{N}}{{\rm{a}}}_{2}{\rm{O}}+0.93{{\rm{K}}}_{2}{\rm{O}}$$

## The results

### Phase composition and calcium carbonate content

To determine the usefulness of the analyzed suspensions for sequestration of CO_2_ through mineral carbonation, the thermogravimetric and differential analyses were carried out.

In order to confirm the occurrence of mineral carbonation processes, and thereby CO_2_ binding by fly ashes-water suspensions, their phase composition was analyzed, and the content of calcium carbonate, the main product of carbonation, was determined.

**Figure 2:**
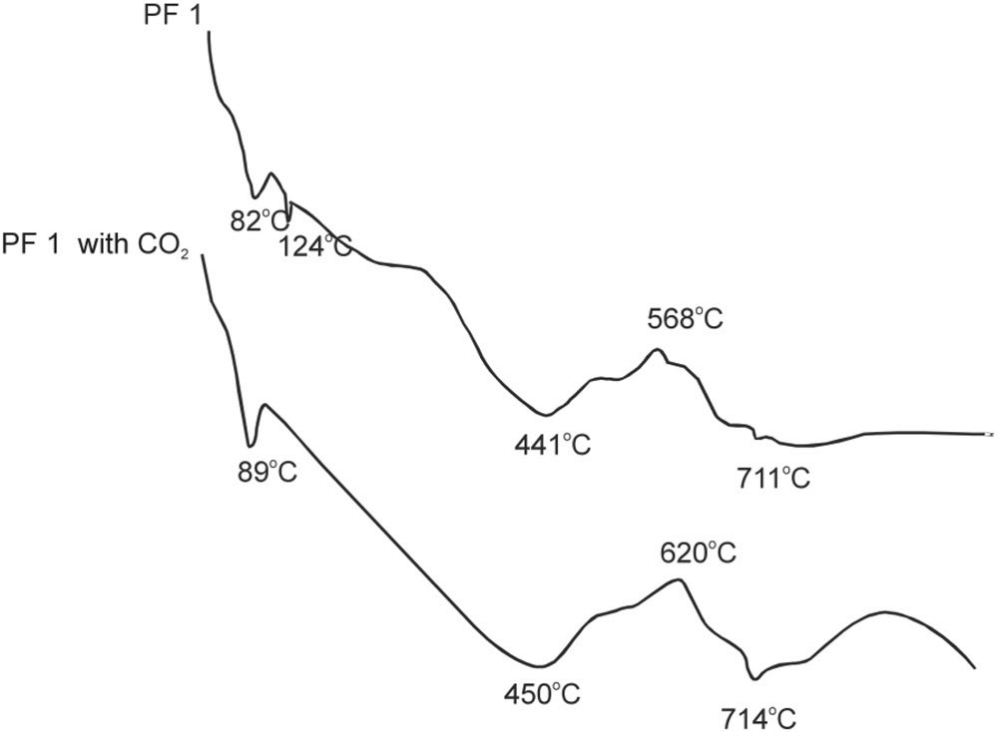
DTA curves for suspensions of PF1 fly ashes: clean (PF 1) and treated with CO_2_ (PF 1 with CO_2_).

The DTA curves of suspensions with FBC fly ashes PF 1 (Fig. 2) show four distinct endothermic effects with the maximums at:

- 82 °C, which can be attributed to the presence of ettringite,
- 124 °C, characteristic for the hydrated phases (silicates, aluminates, and calcium sulfoaluminates),
- 441 °C associated with Ca(OH)_2_,
- 711 °C associated with decomposition of calcium carbonate and the exothermic effect with the maximum at 568 °C, probably associated with the presence of unburned carbon.

The analysis of suspensions of PF 1 fly ashes treated with CO_2_ has not shown the presence of an endothermic effect associated with the presence of silicates, aluminates, and calcium sulfoaluminates. As can be seen from the DTA curves (Fig. 2), there is a very slight decrease in the content of Ca(OH)_2_ and a slight increase in the effect associated with the decomposition of CaCO_3_ for suspensions treated with CO_2_, which confirms the low degree of carbon dioxide binding.

**Figure 3:**
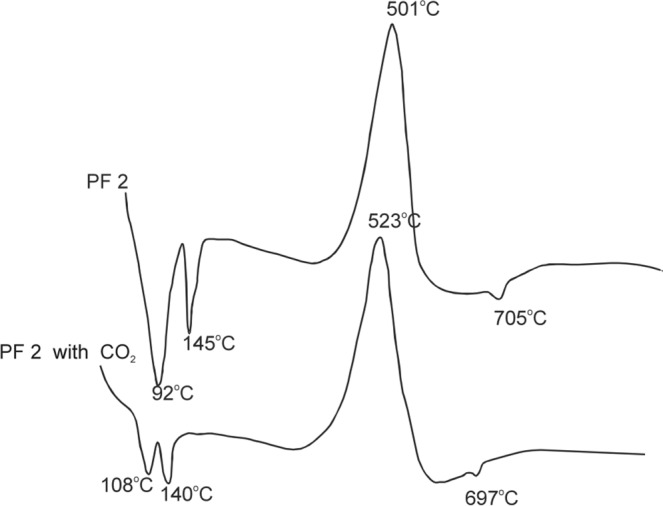
DTA curves for suspensions of PF 2 fly ashes: clean (PF 2) and treated with CO_2_ (PF 2 with CO_2_).

Based on the DTA curves (Fig. 3), the following phases were found in suspensions with PF 2 fly ashes (clean and treated with CO_2_):

- Ettringite and C-S-H (effects with maximums at 92, 108, 145, and 140 °C);
- Unburned carbon (with maximums at 501 and 523 °C)
- Calcium carbonate (with the maximum at 697 and 705 °C).

**Figure 4:**
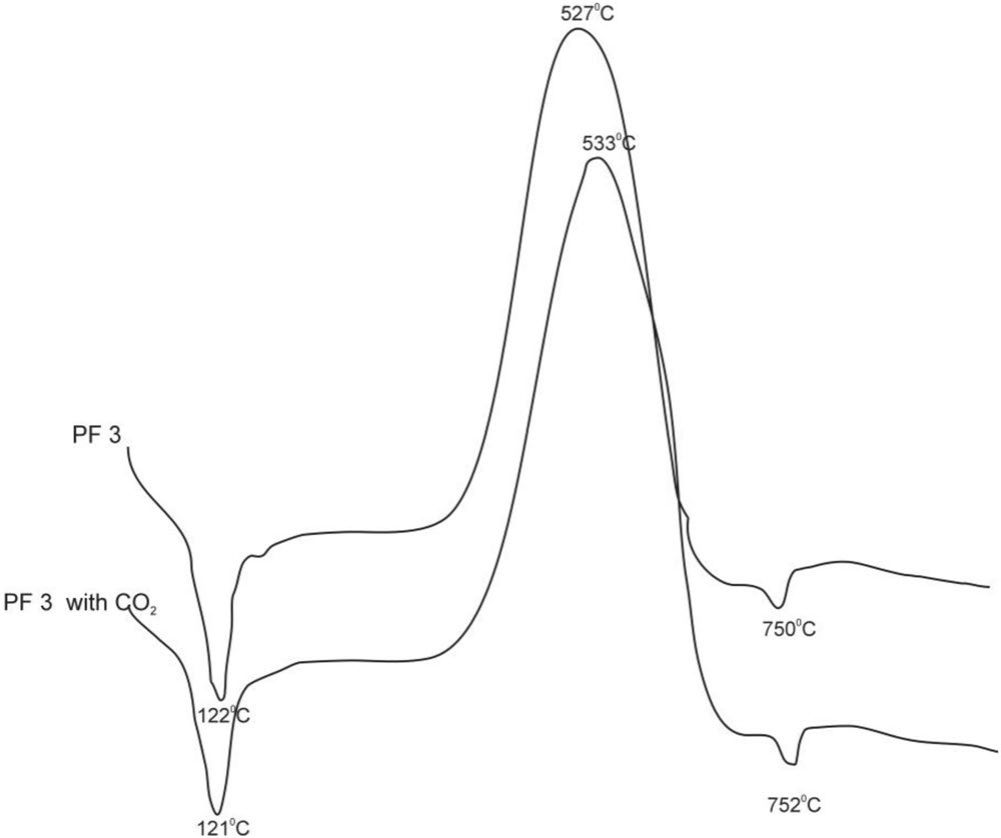
DTA curves for suspensions of PF 3 fly ashes: clean (PF 3) and treated with CO_2_ (PF 3 with CO_2_).

The tested suspensions prepared from PF 3 fly ashes (Fig. 4) are characterized by the presence of the following phases:

- Hydrated phases of silicates, aluminates, and calcium sulfoaluminates (effects with maximums at 121 and 122 °C);
- Unburned carbon (with the maximum at 527 and 533 °C);
- Calcium carbonate (with the maximum at 750 and 752 °C).

All of the analyzed suspensions contain hydrated phases of silicates, aluminates, calcium sulfoaluminates, and calcium carbonate. In addition, suspensions with PF 1 and PF 2 fly ashes contain unburned carbon.

**Figure 5:**
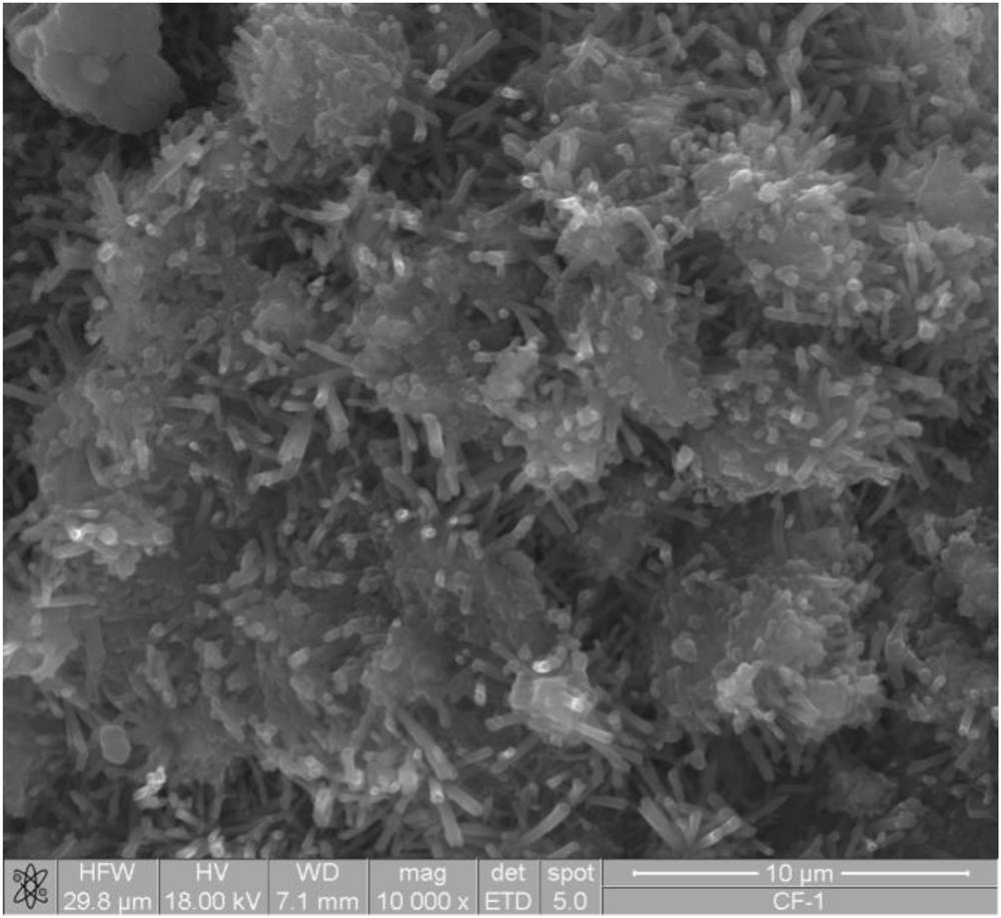
Suspension of PF 2 fly ashes treated with CO_2_ (PF 2 with CO_2_).

The microstructural analysis confirmed the results of thermogravimetric analysis. The image of the PF 2 sample (Fig. 5) is dominated by the hydration products occurring on the surface of ashes particles; the surface is covered with ettringite crystals; visible C-S-H phase.

**Figure 6:**
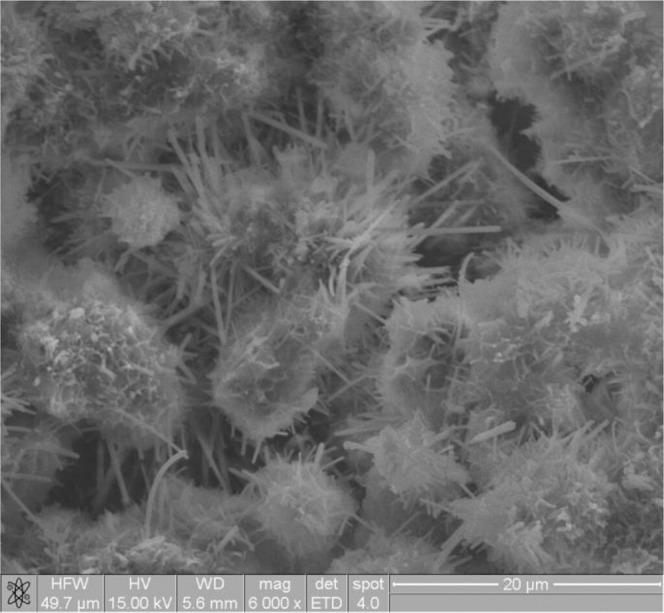
Suspension  of PF 3 fly ashes treated with CO_2_ (PF 3 with CO_2_).

In Fig. 6 (sample PF 3) the surface of the sample is covered with a layer of prismatic ettringite crystals underlain by gel forms of silicates and aluminates.

### Effect of CO_2_ on pollutants leachability

Due to the fact that FBC fly ashes are used primarily in mines in the suspension technology, the obtained results were compared with requirements contained in the PN-G-11011 standard: Materials for Backfilling and Caulking of Cavings – Requirements and Tests (Table 5).

**Table 5:** The effect of carbonation on pollutants leachability in aqueous suspensions in the analysed FBC fly ashes.

Type of designation	Suspensions with FBC fly ashes						Limit values of leachability in the PN-G-11011 standard
	PF 1		PF 2		PF 3		
	clean	with CO_2_	clean	with CO_2_	clean	with CO_2_	
**Element/ion, mg/dm**^**3**^**:**							
Zn	0.0220	0.024	0.037	0.028	0.32	0.046	2.0
Cu	0.0030	0.001	0.00072	0.00026	0.0006	0.00020	0.5
Pb	0.0023	0.0005	0.00005	0.00003	0.00003	0.00001	0.5
Ni	0.0010	0.0008	0.00036	0.00033	0.00023	0.00022	—
As	0.0547	0.0367	0.00142	0.00126	0.0056	0.0044	0.2
Hg	0.0015	0.0014	0.00045	0.00022	0.00048	0.00047	0.02
Cd	0.0006	0.0005	0.0004	0.00008	0.00023	0.00015	0.1
Cr	0.104	0.0690	0.0014	0.0019	0.0071	0.0015	0.2
Cl^−^	12	10	106.4	44.3	9.8	6.2	1,000.0
SO_4_^2−^	250	259	465.3	684.3	528.9	892.3	500.0
ChZT mg O_2_ mg/dm^3^	<5	<5	13.5	52.7	<5	6.3	100
pH	10.2	9.8	10.7	7.9	9.0	8.0	6.0–12.0

Concentrations of pollutants determined by leachability tests mostly do not exceed the limit values specified in the PN-G-11011 standard: Materials for Backfilling and Caulking of Cavings – Requirements and Tests. The only exceptions are sulfates whose value exceeds the limit of 500 mg/dm^3^ (for suspensions with PF 2 and PF 3) and pH values (for PF1 suspension).

A reduction of leachability in the majority of the analyzed pollutants has been observed in all of the analyzed suspensions. The exception is the leachability of SO_4_, which increased for the PF 2 and PF 3 fly ashes.

## Discussion

The degree of CO_2_ binding in the analyzed suspensions was calculated on the basis of thermogravimetric analysis (Table 6, Figs. 1−3) based on the difference in calcium carbonate content using following formula^10^:

$$C{O}_{2uptake}[ \% ]=\frac{{{\rm{CO}}}_{{\rm{2final}}}[ \% ]-{{\rm{CO}}}_{{\rm{2initial}}}[ \% ]}{100-{{\rm{CO}}}_{{\rm{2final}}}[ \% ]}\cdot 100$$

Where: CO_2uptake_− the extent of carbonation, CO_2initials_− initial carbonate content of the sample, CO_2final_− final carbonate content of the sample.

**Table 6:** The content of CaCO_3_ in the test suspensions and the degree of carbonation.

Suspension type	Clean suspension		Subjected to CO_2_		Degree of carbonation [%]
	range of temperatures [°C]	content of CaCO_3_ [%]	range of temperatures [°C]	content of CaCO_3_ [%]	
Suspensions with PF 1	695−900	0.4	695−900	0.7	0.30
Suspensions with PF 2	700−800	0.7	700−800	1.8	1.12
Suspensions with PF3	715−850	4.10	715−850	4.80	0.74

$$C{O}_{2uptake}$$

$${{\rm{CO}}}_{2{\rm{initials}}}$$

$${{\rm{CO}}}_{2{\rm{final}}}$$.

The highest content of CaCO_3_ and the same degree of carbonation has been observed in the case of suspensions with PF 2 fly ashes, while the lowest degree of binding was shown by PF1.

The leachability is the result of interdependent processes^11^. The basic reaction of carbonation, the reaction of Ca(OH)_2_ with carbon dioxide, as a result of which calcite is formed, lowers the pH. In the case of the analyzed fly ashes, it lowers the pH from about 11 to about 8 (Table 5).

The reduction of leachability of Cr and Pb (found in all suspensions) is probably due to the pH change, which after carbonation is close to the pH of the solution with the minimum solubility of Cr and Pb. The leachability is also reduced by the sorption of metals in the newly formed minerals. They can also form complexes with iron and aluminium hydroxides and oxides. The reduction of leachability of Pb and Cr ions may also be caused by the formation of new oxides or sulfates^12^.

In the case of the reduction of leachability of Zn, Cr, and Pb, recorded for all analyzed suspensions based on the presence of C-S-H, it can be explained by their immobilization by C-S-H^13,14^.

The increase in leachability of SO_4_^2−^ (suspensions of PF 1, PF 2, and PF 3) may be due to carbonation which causes decomposition of ettringite and results in the formation of well-soluble CaSO_4_^15^.

The reduced leachability of arsenic is explained by adsorption and coprecipitation to form solid solution with calcite^16^. The reduction of Cu leachability is explained by copper carbonate formation^17^.

In the case of carbonation, an important factor in lowering leachability of some heavy metal ions (Cd, Zn, Mn, Co, Ni, Pb, or Sr) and ions of SO_4_^2−^ is their sorption on calcite, leading to coprecipitation^18,19^.

## The use of FBC fly ashes in mining ‒ potential for sequestration

The mineral sequestration of CO_2_ using FBC fly ashes from hard coal is a particularly interesting option for southern Poland, where the potential for geological storage is limited^20^.

FBC fly ashes from hard coal combustion have been used in Poland for many years, mainly in mining, construction materials, and road construction^4^.

According to estimates from 2013, the amount of CO_2_ that can be disposed of using a mixture of fly ashes and solid waste from calcium flue gas desulfurization ‒ FBC fly ashes, assuming the use of waste already used in the mining industry and unused waste, is 11.3 Gg of CO_2_ per year^21^.

In 2016, 1,142,733 Mg of by-products of combustion were used in coal mines, of which 1,129,795 Mg were fly ashes (99.87%). It is estimated that 1,122,000 tons of by-products of combustion will be used in 2020^22^. Assuming that the FBC fly ashes will be half of the by-products of combustion used in underground mining, i.e. 561,000 tons, the average potential amount of bound CO_2_ for the maximum binding capacity of CO_2_ will constitute 6,844.2 tons (12.2%), while the average maximum binding capacity of CO_2_ will amount to 5,329.5 tons (9.5%).

Taking into account the degree of carbonation, a maximum of 1,683 tons of CO_2_ can be bound for the degree of binding of 0.3 and 6,171 tons of CO_2_ for the degree of binding of 1.1.

## Conclusions

Fluidized bed combustion (FBC) fly ashes have high content of CaO and free CaO, which makes them a good candidate for CO_2_ binding through mineral carbonation.

The studies confirmed the occurrence of the carbonation process. The main product of the mineral carbonation process is calcite. An additional effect is lowering the pH in the course of the process. It has been confirmed that the carbonation process affects the leachability of pollutant. The highest degree of binding was shown by PF 2 fly ashes. In all cases, the presence of hydrated silicates, aluminates, and calcium sulfoaluminates has been confirmed.

Fluidized bed combustion (FBC) fly ashes are also interesting material for CO_2_ binding because they are used in mines (suspension technology). The large-scale use of the suspension technology in the Polish underground mining may make it feasible to use it for CO_2_ binding, because of mines’ enormous experience^5,6^. This solves the problem of utilization of carbonation products. This way the waste will be managed and CO_2_ emission reduced.
